# Translating evidence into practice: eligibility criteria fail to eliminate clinically significant differences between real-world and study populations

**DOI:** 10.1038/s41746-020-0277-8

**Published:** 2020-05-11

**Authors:** Amelia J. Averitt, Chunhua Weng, Patrick Ryan, Adler Perotte

**Affiliations:** 1 Department of Biomedical Informatics, Columbia University, New York, NY USA; 2 Janssen Research and Development, Titusville, NJ USA

**Keywords:** Communication and replication, Epidemiology, Epidemiology, Outcomes research, Randomized controlled trials

## Abstract

Randomized controlled trials (RCTs) are regarded as the most reputable source of evidence. In some studies, factors beyond the intervention itself may contribute to the measured effect, an occurrence known as heterogeneity of treatment effect (HTE). If the RCT population differs from the real-world population on factors that induce HTE, the trials effect will not replicate. The RCTs eligibility criteria should identify the sub-population in which its evidence will replicate. However, the extent to which the eligibility criteria identify the appropriate population is unknown, which raises concerns for generalizability. We compared reported data from RCTs with real-world data from the electronic health records of a large, academic medical center that was curated according to RCT eligibility criteria. Our results show fundamental differences between the RCT population and our observational cohorts, which suggests that eligibility criteria may be insufficient for identifying the applicable real-world population in which RCT evidence will replicate.

## Introduction

Generalizability closes the gap between biomedical research and clinical practice^1^. When research is translated into the healthcare setting, the application of biomedical evidence to clinical care is known as evidence-based medicine (EBM). Since its inception in the 1990s, EBM has become the standard of operation for many clinicians^2–5^. EBM encourages clinicians to seek the most reputable evidence for any patient, according to a hierarchy of study quality in which randomized controlled trials (RCTs) are the best single study design^5^. RCTs are most often used to unbiasedly assess the effect of an intervention, such as a drug or procedure, on an outcome.

Although EBM may be employed successfully for many different clinical decisions, challenges remain. Underlying EBM’s success is the assumption that the effect shown in RCTs will replicate in real-world populations^6,7^. However, research has shown that factors beyond the intervention itself, such as age, sex, or medical history, may modify the measured effect, a phenomenon known as heterogeneity of treatment effect (HTE)^8^. If the RCT population differs from the real-world population based on factors that induce HTE, RCT results will not be replicated in real-world application. Realistically, clinicians cannot evaluate HTE on a case-by-case basis and must assume that HTE is not a significant factor. However, when applying evidence from RCTs, this assumption is likely unmet. Research has shown that HTE is often found to exist^9,10^. This raises concerns for reproducibility of studies in the presence of additional heterogeneity in real-world populations.

The RCT is well-regarded for many reasons, but randomization is the most important. Randomization ensures the highest possible internal validity, which speaks to whether the true effect is biased by systematic error^11,12^. The notion of internal validity does not speak to how well the causal relationship will generalize, only how unbiased it is for the study population. The patients for which the effect estimate is internally valid are nominally defined by eligibility criteria. These criteria both stipulate the characteristics that all study patients must share and nominally identify the real-world population for which the effect is internally valid. When operationalized, the eligibility criteria are represented as inclusion and exclusion criteria^13–15^; and with every addition of a criterion to a study population, a different sub-population is identified with increasingly controlled conditions^16^. If HTE exists, then application of eligibility criteria to a population may identify a sub-population of patients for which there is a more homogeneous effect of the intervention.

RCTs often employ very restrictive eligibility criteria and are often cited as poorly representative of the real-world, as many subpopulations may be excluded. This may result in poor external validity. External validity refers to the extent to which the treatment effect estimate applies those outside of the study with potentially different patient and treatment setting characteristics^11^. External validity always poses a concern, except in the circumstance in which HTE is known to be absent.

With poor external validity, replication of the study effect can be challenging^17–21^. Replication of trial evidence with real-world data, ideally, requires that the right persons, in the right treatment setting, exist in the right proportions. In the context of treating a population that differs significantly from the clinical trial population, it can be unclear how appropriate the evidence is for this new population. Presumably, the eligibility criteria of a study should be sufficient to identify the population in which the effect will replicate, which we call the applicable population.

To address this knowledge gap, we leverage observational data to assesses if RCT populations and real-world populations after application of eligibility criteria differ. If the populations differ, the evidence may not apply due to HTE. If HTE exists in observational populations, it may impede the replication of RCT effect estimates. These methods will contribute (i) a means to determine if the eligibility criteria are adequate for identifying the applicable population; (ii) a framework for evaluating the external validity of studies; and (iii) highlight tensions between assumptions of EBM practice and qualities of reputable evidence.

This research may encourage clinicians to reconsider the assumptions made when practicing EBM, and whether these assumptions are valid. Furthermore, the empirical evidence put forth by this study highlights the limitations of the current system of clinical knowledge generation. The current system sacrifices external validity in favor of internal validity, through the selection of the experimental population. Such a decision impedes the ability of experimental evidence to translate to the general population, resulting in non-optimal or damaging clinical care. This problem motivates the use of study populations that are more representative of the real-world and is only truly optimized when study populations and the populations targeted for treatment are one in the same. Such an analysis is called real-world evidence (RWE) generation, in which clinical knowledge is learned from the analysis of routinely collected, real-world data^22^. The results of this research identify the need for RWE in clinical medicine and underscore how RWE may improve the practice of EBM.

## Results

### Experimental vs. observational populations

**Fig. 1:**
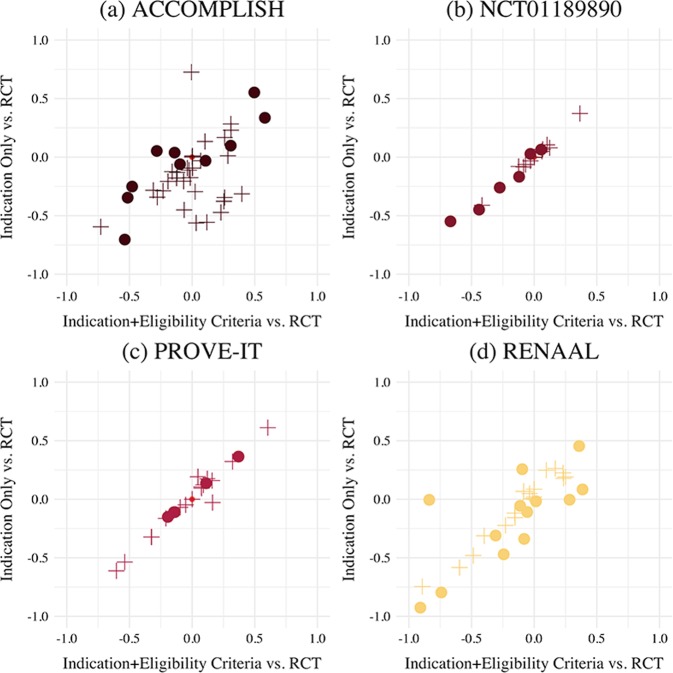
Summary of *∆*_RCT_ for baseline characteristics of Indication Only vs RCT and ∆_RCT_ Indication + Eligibilty Criteria vs. RCT. **a** ACCOMPLISH trial **b** NCT01189890 trial (sitagliptin vs. glimepiride), **c** PROVE-IT trial **d** RENAAL trial. The shape of the marker corresponds to the data type. Circles (●) denote the standardized difference in the mean of continuous data. Pluses (**+**) denote the difference in percentage points of discrete data.

The results of this study are presented in Tables 1–4 and Fig. 1.

**Table 1:** Results for sitagliptin vs. glimepiride trial.

	Sitagliptin vs. glimepiride *Hartley*^42^				Columbia University Irving Medical Center (CUIMC)			
Baseline charactertics	Sitagliptin	Glimepiride	Pooled		Indication only		With eligibility criteria	
	*n* = 197	*n* = 191	*n* = 388	*σ*	*n* = 5942	Δ_RCT_	*n* = 3056	Δ_RCT_
Age	70.6	70.8	70.7	4.85	69.03	−0.260^†^	68.98	−0.275
Sex								
Male	93	77	43.8%		35.87%	−0.079	31.41%	−0.124
Female	104	114	56.2%		64.11%	0.079	68.55%	0.124
Unknown	0	0	0.0%		0.02%	0.000	0.03%	0.000
Race								
White	121	103	57.7%		16.62%	−0.411	16.10%	−0.416
Multi-racial	48	61	28.1%		33.03%	0.049	34.29%	0.062
Native American/Alaska Native	18	15	8.5%		0.09%	−0.084	0.07%	−0.084
Asian	5	12	4.4%		1.17%	−0.032	1.44%	−0.029
African American	4	0	1.0%		11.51%	0.105	11.32%	0.103
Native Hawaiian/Pacific Islander	1	0	0.3%		0.35%	0.001	0.29%	0.000
Unknown	0	0	0.0%		37.23%	0.372	36.49%	0.365
Body weight	76.9	75.3	76.11		76.81	0.028	75.39	−0.030
BMI	29.7	29.7	29.7	4.54	30.35	0.064	30.19	0.055
Duration of DM (years)	8	9.4	8.69	6.43	3.97	−0.549	3.30	−0.668
HbA1c % mean	7.8	7.8	7.8	0.7	7.52	−0.167	6.81	−0.120
Min	6.4	5.7	6.06		3.87	−1.305	4.29	−1.059
Max	10.6	9.9	10.25		15.8	3.307	15.8	3.3301
HbA1c								
<8.0%	131	125	66.0%		59.61%	−0.064	59.00%	−0.070
≥8.0%	66	66	34.0%		33.74%	−0.003	34.20%	0.002
Unknown	0	0	0.00%		6.65%	0.066	6.81%	0.068
FPG	168.4	169.7	169.04	33.21	140.35	−0.448	141.55	−0.440

*Δ*_RCT_ = difference from observational cohort and reported RCT data; standardized difference in the means for continuous variables; difference in percentage points for discrete variables.

*BMI* body mass index, *DM* diabetes mellitus, *yrs* years, *HbA1c* hemoglobin A1c, *Min* minimum, *Max* maximum, *FPG* fasting plasma glucose.

**Table 2:** Results for atorvastatin vs. pravastatin trial (PROVE-IT).

	The PROVE-IT Trial Cannon et al.^41^				Columbia University Irving Medical Center (CUIMC)			
Baseline charactertics	Pravastatin	Atorvastatin	Pooled		Indication only		With eligibility criteria	
	*n* = 2063	*n* = 2099	*n* = 4162	*σ*	*n* = 3972	Δ_RCT_	*n* = 3180	Δ_RCT_
Age	58.3	58.1	58.20	11.25	60.37	0.137	59.95	0.111
Sex								
Male	1617	1634	78.11%		45.92%	−0.322	45.88%	−0.323
Female	445	465	21.89%		54.08%	0.322	54.09%	0.323
Unknown			0.00%		0.03%	0.000	0.03%	0.000
Race								
White	1865	1911	90.73%		28.23%	−0.611	71.42%	−0.604
Other	198	188	9.27%		71.77%	0.611	28.58%	0.604
Diabetes	361	373	17.64%		29.82%		26.57%	
Hypertension	1014	1077	50.24%		60.72%	0.105	57.64%	0.074
Current smoker	766	763	36.74%		4.48%	−0.323	4.18%	−0.326
Prior MI	395	374	18.48%		34.42%	0.159	34.40%	0.159
PCI								
Prior to index event	320	322	15.43%		10.31%	−0.048	10.31%	−0.051
After index event	1426	1442	68.91%		15.30%	−0.536	15.16%	−0.538
Coronary bypass surgery	221	233	10.91%		4.00%	−0.069	1.38%	−0.095
Peripheral artery disease	136	105	5.79%		15.17%	0.094	13.33%	0.075
Prior statin therapy	514	535	25.20%		42.73%	0.175	37.30%	0.121
Index event								
Unstable angina	614	604	29.26%		48.47%	0.192	50.88%	0.046
MI without ST segment elevation (NSTEMI)	757	747	36.14%		19.80%	−0.163	15.22%	−0.209
MI with ST segment elevation (STEMI)	690	748	34.55%		31.73%	−0.028	33.90%	0.163
Median lipid values								
Total cholesterol	180	181	180.50	–	171.67	−0.151	169.54	−0.194
LDL cholesterol	106	106	106.00	–	100.41	−0.110	99.18	−0.138
HDL cholesterol	39	38	38.50	–	45.07	0.364	45.06	0.370
Triglycerides	154	158	156.02	–	141.95	−0.110	137.95	−0.145

**Table 3:** Results for losartan vs. placebo trial (RENAAL).

	The RENAAL Trial Brenner et al.^39^				Columbia University Irving Medical Center (CUIMC)			
Baseline characteristics	Losartan	Placebo	Pooled		Indication only		With eligibility criteria	
	*n* = 751	*n* = 762	*n* = 1513	*σ*	*n* = 3818	Δ_RCT_	*n* = 72	Δ_RCT_
Age	60	60	60.00	7.00	63.72	0.257		−0.095
Sex								
Male	462	494	63.19%		40.86%	−0.223	40.28%	−0.229
Female	286	268	36.62%		59.11%	0.225	59.72%	0.231
Unknown	0	0	0.00%		0.03%	0.000	0.00%	0.000
Race								
Asian	117	135	16.66%		0.58%	−0.157	0.00%	−0.153
Black	125	105	15.20%		15.82%	0.006	13.89%	−0.013
White	358	378	48.65%		0.92%	−0.481	1.39%	−0.486
Hispanic	140	136	18.24%		36.14%	0.179	41.67%	0.234
Other	11	8	1.26%		27.50%	0.262	18.06%	0.168
Unknown	0	0	0.00%		19.04%	0.190	25.00%	0.250
BMI	30.0	29	29.50	6.00	30.56	0.084	34.00	0.386
Blood pressure (mmHg)								
Systolic	152.0	123	137.39	19.50	136.95	−0.017	137.78	0.015
Diastolic	82.0	82	82.00	10.50	71.01	−0.796	71.94	−0.741
Mean arterial	105.5	106	105.75	11.25	104.01	−0.109	104.86	−0.055
Pulse	69.4	70.8	70.11	17.75	79.65	0.454	77.56	0.359
Medical history								
Use of antihypertension drugs	693	721	93.46%		18.91%	−0.745	4.17%	−0.893
Angina pectoris	65	75	9.25%		14.14%	0.049	5.56%	−0.037
Myocardial infarction	75	94	11.17%		17.89%	0.067	2.78%	−0.084
Coronary revasc.	1	1	0.13%		2.02%	0.019	0.00%	−0.001
Stroke	0	1	0.07%		8.64%	0.086	0.005	−0.001
Lipid disorder	234	271	33.38%		58.15%	0.248	43.06%	0.097
Amputation	65	69	8.86%		1.60%	−0.068	0.00%	−0.089
Neuropathy	375	397	51.02%		19.83%	−0.312	11.11%	−0.399
Retinopathy	494	470	63.71%		5.40%	−0.583	4.17%	−0.595
Current smoking	147	130	18.31%		6.47%	−0.118	2.78%	−0.155
Laboratory values								
Median urinary alb:creat ratio	1237	1261	1249.09		NED		NED	-
Serum creatinine (mg/dL)	1.9	1.9	1.90	0.50	1.89	−0.004	2.45	0.282
Serum cholsterol (mg/dL)								
Total	227	229	228.01	55.50	164.98	−0.926	171.11	−0.908
LDL	142	142	142.00	45.99	132.18	−0.005	98.99	−0.837
HDL	45	45	45.00	15.50	43.86	−0.056	43.02	−0.112
Serum triglycerides (mg/dL)	213	225	219.04	190.07	154.29	−0.310	156.21	−0.308
Hemoglobin	12.5	12.5	12.50	1.85	11.53	−0.470	11.92	−0.243
Glycosylated hemoglobin (%)	8.5	8.4	8.45	1.65	8.35	−0.339	8.24	−0.080

**Table 4:** Results for benazepril-amlodipine vs. benazepril and hydrochlorothiazide (HCTZ) trial (ACCOMPLISH).

	The ACCOMPLISH Trial NEJM^40^				Columbia University Irving Medical Center (CUIMC)			
Baseline characteristics	Benazepril-amlodipine	Benazepril– HCTZ Group	Pooled		Indication only		With eligibility criteria	
	*n* = 5744	*n* = 5762	*n* = 11,506	*σ*	*n* = 36,854	Δ_RCT_	*n* = 4198	Δ_RCT_
Age								
≥65 years	3813	3827	66.40%		17.98%	−0.451	60.05%	−0.063
≥70 years	2363	2340	40.87%		9.59%	−0.295	43.22%	0.023
Gender								
Female	2296	2246	39.48%		67.81%	0.283	70.41%	0.309
Male	3448	3515	60.52%		32.18%	−0.283	29.56%	−0.310
Unknown	0	0	0.00%		0.01%	0.000	0.02%	0.000
Race								
White	4817	4795	83.54%		25.31%	−0.595	10.65%	−0.729
Black	697	719	12.31%		14.38%	0.010	12.51%	0.002
Hispanic	300	323	5.41%		30.25%	0.230	36.45%	0.310
Other	230	247	4.15%		19.41%	0.167	30.12%	0.260
Unknown	0	0	0.00%		7.25%	0.134	10.26	0.103
Weight	88.7	88.5	88.60	18.95	78.01	−0.346	74.65	−0.514
Waist circumference	103.9	103.8	103.85	15.30	NED	–	NED	–
Body mass index	31	31	31.00	6.20	30.13	−0.061	29.95	−0.096
Blood pressure								
Systolic	145.3	145.4	145.35	18.25	129.75	−0.704	133.41	−0.537
Diastolic	80.1	80.1	80.10	10.75	76.78	−0.251	73.85	−0.479
Pulse	70.5	70.3	70.40	11.00	79.33	0.552	77.95	0.496
eGFR	78.9	79	78.95	21.35	NED*	–	NED*	–
Serum values								
Creatinine (mg/dL)	1.00	1.00	1.00	0.30	1.08	0.098	1.33	0.308
Glucose (mg/dL)	127.9	127.0	127.45	46.60	149.55	0.336	165.77	0.581
Potassium (mmol/L)	4.3	4.3	4.30	0.40	4.28	−0.031	4.36	0.107
Total cholesterol (mg/dL)	184.9	184.1	184.50	39.90	187.36	0.053	168.80	−0.282
HDL (mg/dL)	49.6	49.5	49.55	14.10	50.31	0.038	46.87	−0.140
Previous AHT treatments								
0	169	153	2.80%		75.42%	0.726	2.28%	−0.006
1	1312	1279	22.52%		10.10%	−0.124	6.60%	−0.159
2	2116	2047	36.18%		7.38%	−0.288	12.97%	−0.232
≥3	2147	2283	38.50%		7.11%	−0.314	78.21%	0.397
Lipid lowering agents	3851	3971	67.98%		12.31%	−0.557	79.75%	0.118
Beta blockers	2675	2807	47.64%		13.18%	−0.345	73.56%	0.259
Antiplatlet agents	3710	3735	64.71%		17.48%	−0.472	87.71%	0.230
Characteristics								
Previous MI	1337	1372	23.54%		2.98%	−0.206	16.76%	−0.068
Previous Stroke	762	736	13.02%		1.94%	−0.111	10.53%	−0.025
Previous hospitalization for unstable angina	653	671	11.51%		2.12%	−0.094	11.78%	0.003
Diabetes mellitus	3478	3468	60.37%		22.68%	−0.377	85.76%	0.254
Renal disease	352	353	6.13%		7.25%	0.011	34.69%	0.286
eGFR < 60	1047	1030	18.05%		0.47%	−0.176	16.59%	−0.015
Previous coronary revasc.	2044	2073	35.78%		1.56%	−0.342	7.99%	−0.278
Coronary artery bypass grafting	1248	1197	21.25%		0.53%	−0.207	1.98%	−0.193
Percutaneous coronary intervention	1055	1123	18.93%		1.08%	−0.179	6.52%	−0.124
Left ventricular hypertrophy	763	758	13.22%		0.21%	−0.130	1.32%	−0.119
Current smoking	641	658	11.29%		1.87%	−0.094	7.48%	−0.038
Dyslipidemia	4221	4319	74.22%		18.01%	−0.562	77.45%	0.032
AFib	376	403	6.77%		3.67%	−0.031	13.63%	0.069

NED = not enough data for measurement.

NED* = eGFR is incomplete in a biased manner due to lack of reporting of values greater than 60.

### Sitagliptin vs. glimepiride

The sitagliptin vs. glimepiride trial in elderly patients with Type 2 Diabetes Mellitus is given in Table 1. Application of eligibility criteria to the Indication Only cohort identified the Indication + Eligibility criteria cohort that was more similar to the RCT with regard to BMI, Fasting plasma glucose, and HbA1c % (mean); and less similar to the RCT with regard to age, years since diabetes diagnosis, gender, HbA1c > 8%, race/ethnicity, and weight. Indication + Eligibility Criteria patients did not significantly differ from the trial in regards to BMI, weight, and HbA1c % (mean), all other baseline characteristics metrics did significantly differ.

These results highlight that the indicated real-world population and the real-world population that meets the stringent eligibility criteria have generally less progressed diabetes than those patients in the trial. This is exemplified by (i) years since diabetes diagnosis, which is 3.97 for the Indication Only cohort and 3.30 in the Indication + Eligibility Criteria cohort, but is 8.69 in the trial (*p* = 0.007) and (ii) fasting plasma glucose, which is 140.35 in the Indication Only cohort and 141.55 in the Indication + Eligibility Criteria cohort, but is 169.04 in the trial (*p* = 0.007). With regard to these two baseline characteristics metrics, the application of the eligibility criteria to the Indication Only cohort identified a subset of patients with a fasting plasma glucose that was more similar to the trial and a years since diabetes diagnosis that was less similar to the trial.

### PROVE-IT

The atorvastatin vs. pravastatin trial in patients with a history of ACS (PROVE-IT Trial) is given in Table 2. Application of eligibility criteria to the Indication Only cohort identified the Indication + Eligibility Criteria cohort that was more similar to the RCT with regard to age, race/ethnicity, diabetes, hypertension, prior MI, peripheral artery disease, and prior statin therapy, and less similar to the RCT with regard to sex, current smoker, percutaneous coronary intervention, index event, and median lipid values. Indication + Eligibility Criteria patients differed significantly from the trial in regards to all baseline characteristics.

The results for this trial show that patients that meet either the Indication or the Indication subject to all criteria, have less severe cardiovascular lipid measurements than patients in the trial. This is demonstrated in the median lipid values, where in total cholesterol, LDL, HDL, and triglycerides are 171.67, 100.41, 45.07, and 141.95, respectively, in the Indication Only cohort and 169.55, 99.19, 45.07, and 138.00, respectively, in the Indication + Eligibility Criteria. This is compared to the 180.50, 106.00, 38.50, and 156.02, respectively, that is reported in the trial.

### RENAAL

The losartan vs. placebo trial in patients with diabetic nephropathy (RENAAL Trial) is given in Table 3. Application of eligibility criteria to the Indication Only cohort identified the Indication + Eligibility Criteria cohort that was more similar to the RCT with regard to age, pulse, angina pectoris, coronary revascularization, stroke, lipid disorder, total cholesterol, serum triglycerides, hemoglobin, and glycosylated hemoglobin, and less similar to the RCT with regard to sex, race/ethnicity, blood pressure measurements, use of antihypertensive drugs, myocardial infarction, amputation, neuropathy, retinopathy, current smoking, laboratory values, LDL and HDL. Indication + Eligibility Criteria patients significantly differ from the trial in regards to angina pectoris, stroke, amputation, lipid disorder, glycosylated hemoglobin % all other baseline characteristics metrics significantly differ. Significance of median urinary alb:creatinine ratio measurements could not be assessed due to insufficient reporting in the EHR.

Similar to the trial results previously mentioned, patients enrolled in the RCT demonstrate hallmarks of advanced disease. A greater proportion of trial patients had a medical history of amputation (8.86%), neuropathy (51.02%), and retinopathy (63.71%), than compared to either the Indication Only cohort (1.60%, 19.83%, 5.40%, respectively) or the Indication + Eligibility Criteria cohort (0.00%, 11.11%, 4.17%).

### ACCOMPLISH

The benazepril-amlopidine vs. benazepril-hydocholorothiazide trial in patients with systolic hypertension (ACCOMPLISH Trial) is given in Table 4. Application of eligibility criteria to the Indication Only cohort identified the Indication + Eligibility Criteria cohort that was more similar to the RCT with regard to age, potassium, lipid lowering agents, beta blockers, antiplatelet agents; history of MI, stroke, hospitalization for unstable angina, diabetes mellitus, eGFR < 60, coronary revascularization, CABG, PCI, left ventricular hypertrophy, current smoking, dyslipidemia, and AFib, and less similar to the RCT with regard to sex, race/ethnicity, weight, blood pressure measurements, pulse, creatinine, glucose, total cholesterol, HDL, and history of renal disease. Indication + Eligibility Criteria patients significantly differ from the trial in regards to all baseline characteristics, except for history of previous hospitalization for unstable angina. Significance of waist circumference and eGFR could not be assessed due to data availability and insufficient reporting in the EHR.

The results of the four trials are summarized in Fig. 1. In this figure, each quadrant of the plot corresponds to a trial. For each trial, the Δ_RCT_ for baseline characteristics are plotted for Indication Only vs. RCT and indication + Eligibility Criteria vs. RCT. The minimum and maximum HbA1c measurements for the NCT01189890 trial were excluded in this plot due to biologically implausible values that were likely transcription errors.

## Discussion

This research suggests that eligibility criteria are insufficient for identifying the applicable real-world population in which experimental treatment effects will replicate with confidence. The comparison between the trial and the Indication + Eligibility Criteria cohorts highlights that the RCT and real-world cohorts are not similar. This result suggests that the eligibility criteria may not identify the applicable patients if HTE exists.

In some cases, application of the eligibility criteria to the Indication Only cohort encouraged the mean feature to be more like that of the RCT. For example, the distribution of gender in the PROVE-IT trial. However, much more commonly, the application of the eligibility criteria to the Indication Only cohort also results in (i) an exacerbation of the difference between the Indication Only cohort and the RCT, as was seen with gender in the RENAAL trial; or (ii) an over-correction of the bias between the Indication Only cohort and the RCT, as was seen with gender in the ACCOMPLISH trial.

This evaluation, with something as fundamental as gender, demonstrates that the eligibility criteria do not strictly encourage the data to be more like that reported in the RCT baseline characteristics data. Often, the eligibility criteria identified a subset a patient that was less like the trial on certain baseline characteristics. This suggests that the eligibility criteria applied in a different setting may actually increase confounding and introduce new biases in such an analysis. This assertion is additionally supported by the summarization of results in Fig. 1. A clustering of points near the center of the plot (0.0, 0.0) indicates that the observational cohorts differ very little from the RCT. Points that lie on the 45-degree line are indicative of baseline characteristics that are unaffected by the addition of eligibility criteria to the Indication Only. Deviations from this perfect correlation highlight the extent to which application of eligibility criteria encourage or worsen representativeness to the RCT. The sitagliptin vs. glimepiride trial (NCT01189890) and PROVE-IT show the least impact of the RCT criteria. The high linearity of points in these plots suggests that the eligibility criteria do not identify a subset of patients that are meaningfully different from the Indication Only cohort. This is contrary to the ACCOMPLISH and RENAAL trials. In these plots, there is more variance in the distribution of points along the 45-degree line with certain features improving representativeness and others worsening.

The creation of these observational cohorts permits the comparison of real-world populations to summaries of clinical trials. A number of studies have previously examined the of misalignment between experimental populations and real-world populations by quantifying the discrepancy between these two data sources^23–34^. Despite this ongoing conversation regarding the lack of representativeness and generalizability of clinical trials, the relationship between the eligibility criteria and HTE and how they may contribute to poor external validity, remains poorly addressed. This research makes a thorough assessment of these two populations by comparing experimental cohorts with observational cohorts that were curated by carefully operationalizaed of eligibility criteria; it is highly rigorous and encourages confidence in our assessment of the inconsistencies between the trial and the real-world.

We believe our approach is limited in practice because the task is complex and requires many components to line up. Our methods require not only a substantial amount of observational data, but standardization of that data into a common data model. The use of the Observational Health Data Sciences and Informatics’ common data model (OHDSI CDM) in this research facilitated the normalization of medical concepts to a single code and made simplified the definition of the cohorts.

This study contributes a systematic evaluation of cohort characteristics under eligibility criteria. We show that discrepancies may appear as differences in aggregate features in the baseline characteristics. If these features have a meaningful effect on the outcome, the differences or imbalance between the cohorts, may result in confounding of the observational treatment effect. In this circumstance, the RCT evidence would not applicable to this real-world cohort. Computational methods could assist in identifying patients that match the RCT cohort for applicability, and perhaps such methods should be applied given the results shown here.

Experimental trial participants are not only an inherently poor representation of the target population, but this research suggests that factors beyond eligibility criteria may introduce new hidden bias. Furthermore, the importance of HTE and potential for feature imbalance, even under careful cohort curation, highlight the current methodological gap in trial replication. Through our replication efforts, we were also able to articulate a framework of external validity. As noted earlier, external validity refers to the extent to which the trial results can be applied outside of the experimental setting^13^.

Underlying the results of this research is the inherent tension that exists between the practice of EBM and what is regarded as credible evidence. The RCT, which is the most reputable source of biomedical knowledge, employs highly discriminative eligibility criteria that serve to identify a targeted effect of the intervention. This research uses real-world evidence to demonstrate that EBM practitioners cannot reasonably assume that trial participants are representative of real-world eligible patients. This raises the question as whether the RCT evidence is, therefore, applicable. This consideration is complicated by the inability of clinicians to determine who the applicable patients are. This is despite the publication of eligibility criteria, which are often incompletely or insufficiently reported in the modes of evidence most often consumed by clinicians^35^.

This research does have limitations. Most importantly, the trials presented in this research were selected according to a set of criteria that enabled their analysis using the tools described. These criteria included an active intervention and comparator, published eligibility criteria, and ease of operationalization of concepts. The trials that were investigated as part of this research represent common indications. It is possible that the results presented here are specific to trials of common conditions and may not be representative of rare condition trials.

The translation of clinical trial eligibility criteria to operationalized and computable queries may be prone to subjectivity. Although we sought to represent the criteria as unbiasedly as possible and consulted with clinicians to ensure accuracy, there is inherent ambiguity in the criteria themselves, which make perfect RCT representation impossible. Furthermore, information regarding the eligibility criteria may be found within the clinical note, which was not used when constructing the cohorts in this research. Additionally, when subjecting an observational cohort to many criteria, the resultant cohort may become very small, leading to a lack of power for the detection of relevant differences. In our evaluation of the external validity of trials, we compare aggregate metrics rather than a full distribution of features, which would be preferable. This comparison is the best we can do with the data that is available to us. However, such a comparison may fail to capture meaningful differences between the trial and real-world populations, as distributions with greatly differing functional forms may still have similar means.

Lastly, and most notably, experimental data and EHR data are fundamentally different, which makes comparison between these two sources difficult. Though differences to experimental data may be inherent, the EHR houses the information that is available to clinicians at the time when treatment decisions are made. Furthermore, it is a valuable resource for identifying the applicable patients to support the practice of EBM. We believe that discrepancies between experimental data and EHR data are necessary to study so that we may develop methodologies to ensure appropriate applicability at the point of care.

Based on the results of the research presented, the eligibility criteria, that nominally should be sufficient for effect replication, may not actually be sufficient if HTE exists. If HTE exists and the differences we observed in our cohort are common, factors beyond eligibility criteria may be necessary to identify applicable patients. This finding has significant implications on how we create and apply biomedical evidence.

The expectation of EBM is that the population of patients that a single clinician sees, is an applicable population, and will mirror the population in the RCT in all ways, including the distribution of the treatments effect. This assumption does not take into account variation undocumented factors that affect HTE. If factors that induce HTE are not accounted for in the eligibility criteria but exist, a clinician cannot reasonably assume that the treatment effect will be seen in his treated patient population. The discrepancies between experimental and real-world populations that are presented here may be due to a number of sources, including overly restrictive eligibility criteria, insufficient documentation of eligibility criteria, or the self-selection of trial participants. When seeking to rectify this gap and improve generalizability of RCT findings, these issues may be addressed by the relaxation of trial eligibility criteria, a thorough and accurate description of eligibility criteria (perhaps recorded in a codified manner), or the active recruitment of a representative experimental population. Regardless of the source of this discrepancy, until addressed, careful consideration beyond who is eligible for the trial is necessary to determine whether results of a given RCT are an appropriate source of evidence when considering the care of a given patient.

## Methods

### The comparison of experimental and observational populations

We hypothesized that significant baseline characteristic differences exist between clinical trial populations and observational cohorts that meet all eligibility criteria. Such differences could be the source of poor external validity in the presence of HTE. The presence of differences could be confirmed by comparing empirical distributions of features between the RCT data and real-world observational data. However, patient-level RCT data is rarely released, so such as assessment is infeasible for most published RCTs. The best available proxy is to compare the real-world observational cohort to the summary of baseline characteristics of RCTs, as commonly presented in Table 1 of RCT publications. We will refer to these summary statistics as baseline characteristics. The baseline characteristics summarize the baseline demographic and clinical characteristics for each arm of the study^36^. The intent of publishing this table is to describe the clinical trial population in detail and report the similarity of arms in the RCT post-randomization. This data can also be used to evaluate external validity, and by association, replicability^37^. To examine how potential differences between experimental and observational cohorts may contribute to poor replicability, we compared RCT baseline characteristics with the same metrics from observational EHR data.

### Data

Observational clinical data was obtained from the Columbia University Irving Medical Center (CUIMC) clinical data warehouse (CDW). Data elements evaluated in this study include laboratory measurements, diagnosis codes, and medications. This database is comprises predominantly of emergency and inpatient visits with a smaller number of outpatient visits at the hospital’s teaching clinics. The data used for this research was formatted according to the OHDSI (http://www.ohdsi.org) CDM to support downstream interoperability within the OHDSI community and to support replication and extension by OHDSI collaborators. This research was approved by the Columbia University Institutional Review Board. Informed consent was waived as this the research could not practicably be carried out without the waiver. The code to collect and query the data is freely available at https://github.com/ameliaaveritt/Translating_Evidence_Into_Practice.

### Cohort creation

Corresponding to each RCT, observational cohorts were curated from EHR data according to two approaches. The first approach curated based on only the indication of the drug (Indication Only), e.g., diabetes or heart failure. This cohort represents the most basic assessment that clinicians can make when considering a treatment for a patient, per EBM. The second approach curated based on both the indication of the drug and all published eligibility criteria (Indication + Eligibility Criteria). This cohort represents the most thorough assessment that clinicians can make under EBM.

**Fig. 2:**
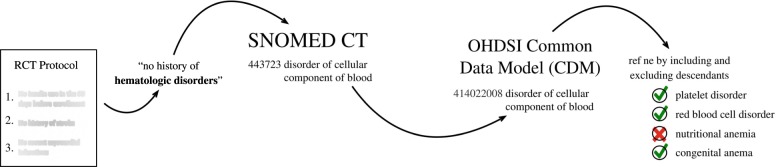
Pipeline to operationalize eligibility criteria using OHDSI tools. The process begins by identifying the resources (e.g., an RCT protocol) that detail the eligibility criteria of a trial. Each criterion is then extracted and mapped to codified concepts in a controlled vocabulary. The concept is then mapped to the OHDSI common data model (CDM), which aggregates the same concepts from different vocabularies, into a single standardized concept. This concept is then refined to best define the eligibility criterion.

Both the Indication Only and Indication + Eligibility Criteria cohorts were constructed using OHDSI’s ATLAS tool. ATLAS is an analytics platform used to support the design and execution of observational analyses. Part of this platform includes the ability to create cohort definitions. Cohort definitions identify a set of patients that satisfy one or more criteria for a duration of time. The Indication Only and Indication + Eligibility Criteria cohorts were defined using this tool. The Indication and Eligibility Criteria that were extracted from published RCT documentation were operationalized using the Observational Medical Outcomes Partnership (OMOP) CDM and served as criteria for cohort definitions. This was a rigorously done procedure, in which medical doctors were consulted to ensure the accuracy of the operationalization and faithfulness to the original criteria. To operationalize the criteria, we created concept sets, which enumerate both the medical concepts that should be included in the definition of our criteria and excludes the concepts that should not be included. This procedure often employed the hierarchical relationships that exist in the OMOP CDM ontology, where in all descendants of a single concept could be selected as part of a concept set and selectively removed, if needed. This procedure is outlined in Fig. 2.

### Cohort comparisons

For each RCT under study, we calculated the pooled baseline characteristics using the metrics reported for both the intervention and comparator arms. Discrete data was summed across both arms and is presented as a percent. Continuous data was taken as the average of each arm’s reported metrics, weighted by the proportion of patients in that arm.

The Indication Only and Indication + Eligibility Criteria cohorts were queried to obtain metrics that corresponds to the RCT baseline characteristics. To explore the differences that exist between the observational patient cohorts and the RCT patient cohort, we calculated (i) the standardized difference in the means for continuous variables and (ii) percentage point differences between discrete variables (Δ_RCT_). If Δ_RCT_ evaluates to zero, this indicates that the observational cohort does not differ from the trial cohort. If Δ_RCT_ does not equal zero, this indicates that observational and trial cohorts differ, with greater magnitudes corresponding to greater discrepancies between the cohorts.

### Trial selection

For this research, we purposefully picked landmark clinical trials, which are highly influential studies that are noted to change the practice of medicine. We began with a list of landmark trials, and after application of criteria that are outlined below, we decided on a small number. Our primary focus was landmark trials, but to increase the diversity of studies and to demonstrate applicability outside of efficacy trials, we evaluated a safety trial that met our criteria as well.

When selecting candidate trials for this research, there were practical considerations that informed our choice of trials^38^. The RCT must have an active intervention and comparator drug, as we would be unable to sufficiently codify a cohort exposed to a placebo. Additionally, the intervention cannot be a new investigatory drug, as it would not exist in our EHR. The eligibility criteria for the RCT must be published and accessible; and most of the eligibility criteria must be hard criteria that are easily operationalized into concept codes (e.g., “age of at least 55 years”). While most trials have inescapable soft criteria that are not easily operationalized (e.g., “no contraindications” or “no current participation in another clinical trial”), it is important that our chosen trials have few of these. Consider, for example, the soft criteria “expected survival of at least 2 years”, which embodies a judgment call by a healthcare practitioner that cannot reasonably replicated with data. Finally, we sought trials that detailed a patient population that exists within the CUIMC EHR. This would ensure that a sufficient number of patients remain in our cohorts after application of the eligibility criteria. As we are interested in comparing the RCT Table 1 metrics with the same metrics from our observational cohort, it is important that our observational data contain as many patients as possible, as greater number of patients will increase confidence that our reported data is truly representative of the CUIMC population.

To that end, we investigated four trials (1) the RENAAL Trial, which compared the effect of losartan and placebo on diabetic nephropathy^39^; (2) the ACCOMPLISH Trial^40^, which compared benazepril-amlodipine to benazepril and hydrochlorothiazide on CV-related mortality, (3) the PROVE-IT Trial^41^, which compared atorvastatin and pravastatin in patients with a history of acute coronary syndrome (ACS); and (4) the sitagliptin and glimepiride trial^42^, which compared sitagliptin and glimepiride in elderly, diabetic patients. RENAAL, ACCOMPLISH, and PROVE-IT are Landmark RCTs with efficacy endpoints, and the sitagliptin vs. glimepiride trial is a smaller trial with a safety endpoint. Details on how the Indication Only and Indication + Eligibility Criteria cohorts were created can be found in the Supplementary Information Tables 1–10.

### Reporting summary

Further information on research design is available in the Nature Research Reporting Summary linked to this article.

## Supplementary information

Reporting Summary

Supplementary Information

## Data Availability

Due to the existence of protected health information, the data are not publicly available. However, due to the standardized nature of our data and the coded vocabulary for our data, external researchers can replicate our work through a network study in the OHDSI consortium where Columbia University serves as the coordinating center.

## Code availability

To analyze the data that was presented in this study, this research implemented custom code. This code is freely available at https://github.com/ameliaaveritt/Translating_Evidence_Into_Practice

## References

[1] Wong, V. C. & Steiner P. M. Replication designs for causal inference. *EdPolicyWorks Working Paper Series. 2018* [cited 2019 Mar 26]. Available from: http://curry.virginia.edu/uploads/epw/62_Replication_Designs.pdfhttp://curry.virginia.edu/edpolicyworks/wp

[2] Djulbegovic, B. & Guyatt, G. H. Progress in evidence-based medicine: a quarter century on. *Lancet* **390**, 415–423 (2017). 10.1016/S0140-6736(16)31592-6 28215660

[3] Djulbegovic, B. & Guyatt G. in *Users Guide to Medical Literature*. 3rd edn. (McGraw-Hill Education, 1976).

[4] Djulbegovic, B., Guyatt, G. H. & Ashcroft, R. E. Epistemologic inquiries in evidence-based medicine. *Cancer Cont.* **16**, 158–168. 10.1177/107327480901600208 19337202

[5] Sackett DL, Rosenberg WM, Gray JA, Haynes RB, Richardson WS (1996). Evidence based medicine: what it is and what it isn’t. BMJ.

[6] Ioannidis JPA (2014). How to make more published research true. PLoS Med..

[7] Contopoulos-Ioannidis DG, Alexiou GA, Gouvias TC, Ioannidis JPA (2008). Life cycle of translational research for medical interventions. Science.

[8] Kent DM (2016). Risk and treatment effect heterogeneity: re-analysis of individual participant data from 32 large clinical trials. Int. J. Epidemiol..

[9] Fredriksson P, Johansson P (2008). Dynamic treatment assignment. J. Bus. Econ. Stat..

[10] Xie Y, Brand JE, Jann B (2012). Estimating heterogeneous treatment effects with observational data. Socio. Methodol..

[11] Campbell DT, Stanley JC (1963). Experimental and Quasi-experimental Designs for Research.

[12] Burns PB, Rohrich RJ, Chung KC (2011). The levels of evidence and their role in evidence-based medicine. Plast. Reconstr. Surg..

[13] Campbell, D. T. & Stanley, J. C. *Handbook of Research on Teaching*. 1–84 (Houghton Mifflin Company, Boston, 1963).

[14] Hyman, R. *Quasi-Experimentation: Design and Analysis Issues for Field Settings*. Vol. 46, 96–97 (Houghton Mifflin, 1982).

[15] Anderson-Cook, C. M. *Experimental and Quasi-Experimental Designs for Generalized Causal Inference.* Vol. 100 (Wiley, 2005).

[16] Velasco, E. in *Encyclopedia of Research Design* (ed. Salkind, N) (SAGE, Thousand Oaks Publications, 2010).

[17] Wales JA, Palmer RL, Fairburn CG (2009). Can treatment trial samples be representative?. Behav. Res. Ther..

[18] Moher D, Jadad AR, Tugwell P (1996). Assessing the quality of randomized controlled trials. Current issues and future directions. Int. J. Technol. Assess. Health Care..

[19] Britton A (1999). Threats to applicability of randomised trials: exclusions and selective participation. J. Heal. Serv. Res. Policy.

[20] Karanis, Y. B., Canta, F. A. B., Mitrofan, L., Mistry, H. & Anger, C. ‘Research’ vs ‘real world’ patients: the representativeness of clinical trial participants. *Ann. Oncol*. (2016) 10.1093/annonc/mdw392.51/2800468/Research-vs-real-world-patients-the

[21] Stuart EA, Bradshaw CP, Leaf PJ (2015). Assessing the generalizability of randomized trial results to target populations. Prev. Sci..

[22] Sherman RE (2016). Real-world evidence — what is it and what can it tell us?. N. Engl. J. Med..

[23] Kennedy-Martin T, Curtis S, Faries D, Robinson S, Johnston J (2015). A literature review on the representativeness of randomized controlled trial samples and implications for the external validity of trial results. Trials.

[24] Badano LP (2003). Patients with chronic heart failure encountered in daily clinical practice are different from the “typical” patient enrolled in therapeutic trials. Ital. Hear. J..

[25] Bosch X (2008). Causes of ineligibility in randomized controlled trials and long-term mortality in patients with non-ST-segment elevation acute coronary syndromes. Int. J. Cardiol..

[26] Collet JP (2003). Enoxaparin in unstable angina patients who would have been excluded from randomized pivotal trials. J. Am. Coll. Cardiol..

[27] Costantino G (2009). Eligibility criteria in heart failure randomized controlled trials: a gap between evidence and clinical practice. Intern. Emerg. Med..

[28] Dhruva SS, Redberg RF (2008). Variations between clinical trial participants and medicare beneficiaries in evidence used for medicare national coverage decisions. Arch. Intern. Med..

[29] Ezekowitz JA (2012). Acute heart failure. Circ. Hear. Fail..

[30] Golomb BA (2012). The older the better: are elderly study participants more non-representative? A cross-sectional analysis of clinical trial and observational study samples. BMJ Open..

[31] Hutchinson-Jaffe AB (2010). Comparison of baseline characteristics, management and outcome of patients with non–ST-segment elevation acute coronary syndrome in versus not in clinical trials. Am. J. Cardiol..

[32] Melloni C (2010). Representation of women in randomized clinical trials of cardiovascular disease prevention. Circ. Cardiovasc. Qual. Outcomes.

[33] Steinberg BA (2007). Global outcomes of ST-elevation myocardial infarction: comparisons of the enoxaparin and thrombolysis reperfusion for acute myocardial infarction treatment-thrombolysis in myocardial infarction study 25 (ExTRACT-TIMI 25) registry and trial. Am. Heart J..

[34] Uijen AA, Bakx JC, Mokkink HGA, van Weel C (2007). Hypertension patients participating in trials differ in many aspects from patients treated in general practices. J. Clin. Epidemiol..

[35] Van Spall HGC, Toren A, Kiss A, Fowler RA (2007). Eligibility criteria of randomized controlled trials published in high-impact general medical journals. JAMA.

[36] Schulz KF, Altman DG, Moher D (2010). CONSORT 2010 Statement: updated guidelines for reporting parallel group randomised trials. BMJ.

[37] Furler J, Magin P, Pirotta M, van Driel M (2012). Participant demographics reported in “Table 1” of randomised controlled trials: a case of “inverse evidence”?. Int. J. Equity Health..

[38] Bartlett VL, Dhruva SS, Shah ND, Ryan P, Ross JS (2019). Feasibility of using real-world data to replicate clinical trial evidence. JAMA Netw. Open..

[39] Brenner BM (2001). Effects of losartan on renal and cardiovascular outcomes in patients with type 2 diabetes and nephropathy. N. Engl. J. Med..

[40] Jamerson, K. et al. Benazepril plus Amlodipine or hydrochlorothiazide for hypertension in high-risk patients. *N. Engl. J. Med.* 359, 2417–2428 (2008) http://www.nejm.org/doi/abs/10.1056/NEJMoa0806182. 10.1056/NEJMoa0806182 19052124

[41] Cannon CP (2004). Intensive versus moderate lipid lowering with statins after acute coronary syndromes. N. Engl. J. Med..

[42] Hartley P (2015). Efficacy and tolerability of sitagliptin compared with glimepiride in elderly patients with type 2 diabetes mellitus and inadequate glycemic control: a randomized, double-blind, non-inferiority trial. Drugs Aging.

