# Supplementary material for: Translating evidence into practice: eligibility criteria fail to eliminate clinically significant differences between real-world and study populations
Source: NPJ Digit Med. 2020 May 11;3:67. doi: 10.1038/s41746-020-0277-8 (PMC7214444; doi:10.1038/s41746-020-0277-8)
Supplement: Supplementary file 2 — Supplementary Information [file 41746_2020_277_MOESM2_ESM.pdf]

SUPPLEMENTARY TABLE 1: Sitagliptin vs Glimepiride, Concept Definitions

		Included Concepts		Excluded Concepts	
Indication	concept	concept code	concept name	concept code	concept name
	Type II Diabetes Mellitus	201826	Type 2 diabetes mellitus		

		Included Concepts		Excluded Concepts	
Eligibility Criteria	concept	concept code	concept name	concept code	concept name
	Type I Diabetes Mellitus	201254	Type 1 diabetes mellitus		
	Liver Disease	194984	Disease of Liver		
	Cardiovascular disease	373503	Transient cerebral ischemia		
		316139	Heart failure		
		374384	Cerebral ischemia		
		375557	Cerebral embolism		
		372924	Cerebral artery occlusion		
		40479625	Atherosclerosis of artery		
		4215140	Acute coronary syndrome		
	Hypertension	3004249	BP systolic		
		3012888	BP diastolic		
	Peripheral Vascular Disease (PVD)	321052	Peripheral vascular disease		
	Triglycerides	3022192	Triglyceride [Mass/Volume] in Serum or Plasma		
	Human Immunodeficiency Virus (HIV)	439727	Human Immunodeficiency virus infection		
	Malignancy/"Certain Cancers"	443392	Malignant neoplastic disease	4300118	Squamous cell carcinoma
				4179980	Malignant basal cell neoplasm of skin
	Hematologic Disorder	443723	disorder of cellular component of blood	4280354	nutritional anemia
	Estimate Glomerular Filtration Rate (eGFR)	3049187	eGFR with normals for non-black		
		3053283	eGFR with normals for black		
	Dipeptidyl peptidase 4 (DPP-4) inhibitors	21600783	Dipeptidyl peptidase 4 (DPP-4) inhibitors		
	Insulin	21600713	Insulins and Analogues		
	GLP-1 memetic	40219409	GLP-1 Receptor Agonist		
	Peroxisome proliferator-activated receptor (PPAR)	4354720	PPAR gamma		
	Kidney Disease	4030518	Renal Impairment		
	Surgical Procedure	4301351	Surgical Procedure		
	Substance Abuse	36903635	Substance-Related Disorders	35809374	Tobacco withdrawal syndrome
				36919133	Tobacco abuse
				4209423	Nicotine dependence
				36919130	Nicotine dependence
				434697	Maternal tobacco abuse

Note that all concept definitions include descendants

SUPPLEMENTARY TABLE 2: Sitagliptin vs Glimepiride, Cohort Creation

Indication	A condition occurrence of Type II Diabetes Mellitus<br>∧<br>Age between 65 and 80<br>∧<br>Continuous observation of at least 365 days before and 0 days after index

Eligibility Criteria	No High Triglycerides	Exactly 0 triglyceride measurements > 600mg/dL[2]
	No Hypertension	Exactly 0 BP Systolic measurements > 140 mmHg[2]<br>∧<br>Exactly 0 BP Diastolic measurements > 90 mmHg[2]
	No HIV	Exactly 0 diagnoses of HIV[1]
	No Type I Diabetes Mellitus	≤ 3 diagnoses of Type I Diabetes Mellitus[2]
	No Surgical Procedures	Exactly 0 Surgical Procedures[3]
	No cardiovascular disease	Exactly 0 diagnoses of CVD[2]
	No Liver Disease	Exactly 0 diagnoses of Liver Disease[2]
	No PVD	Exactly 0 diagnoses of PVD[2]
	No Insulin or GLP-1 use	Exactly 0 drug exposures to Insulin/GLP-1[4]
	No DPP-4 Use	Exactly 0 drug exposures to DPP-4[2]
	No Malignancy/Certain Cancers	Exactly 0 diagnoses of Malignancy or "Certain Cancers"[1]
	No Hematologic Disorders	Exactly 0 diagnoses of Hematologic Disorders[1]
	No Renal Impairment	Exactly 0 diagnoses of Renal Impairment[1]
	No eGFR ≥ 35 mL/min	Exactly 0 eGFR measurements ≥ 35 mL/min[1]
	No Substance Abuse	Exactly 0 diagnoses of a History of Substance Abuse[1]

[1]between all days before and 1 days before index start date
[2]between 365 days before and 1 days before index start date
[4]between 56 days before and 1 day before index start state
[3]between 28 days before and 1 day before index start state

SUPPLEMENTARY TABLE 3: Concept Definitions: PROVE-IT, Indication

		Included Concepts		Excluded Concepts	
	concept	concept code	concept name	concept code	concept name
Indication	Acute Coronary Syndrome	35205182	Angina unstable	4329847	Old myocardial infarction
		4329847	Myocardial infarction	44820861	Acute myocardial infarction of unspecified site, subsequent episode of care
				44832376	Acute myocardial infarction of other specified sites, subsequent episode of care
				44834721	Acute myocardial infarction of other lateral wall, subsequent episode of care
				44832374	Acute myocardial infarction of other inferior wall, subsequent episode of care
				44819697	Acute myocardial infarction of other anterior wall, subsequent episode of care
				44820860	Acute myocardial infarction of inferoposterior wall, subsequent episode of care
				44820859	Acute myocardial infarction of inferolateral wall, subsequent episode of care
				44820858	Acute myocardial infarction of anterolateral wall, subsequent episode of care

Note that all concept definitions include descendants

SUPPLEMENTARY TABLE 4: Concept Definitions: PROVE-IT, Eligibility Criteria

		Included Concepts		Excluded Concepts	
	concept	concept code	concept name	concept code	concept name
Eligibility Criteria	Long Term Lipid-Lowering Therapy	21601853	Lipid Modifying Agents		
	Total Cholesterol	3027114	Cholesterol [Mass/volume] in Serum or Plasma		
	Statin	21601855	HMG CoA reductase inhibitors		
	Lipid Lowering Therapy with Fibric Acid	21601864	Fibrates		
	Lipid Lowering Therapy with Niacin	1517824	Niacin		
	CYP420-34	21601919	Imidazole and triazole derivatives		
	Percutaneous Coronary Intervention	4216130	Percutaneous coronary intervention		
	Coronary Artery Bypass Surgery	37522318	Coronary artery bypass		
	Obstructive Hepatobilliary Disease	35902850	Obstructive bile duct disorders (excl neoplasms)		
	Hepatic Disease	194984	Disease of liver		
	Creatinine Kinease Level	3007220	Creatine kinase [Enzymatic activity/volume] in Serum or Plasma		
	Creatinine Serum	3016723	Creatinine serum/plasma		

Note that all concept definitions include descendants

SUPPLEMENTARY TABLE 5: PROVE-IT, Cohort Creation

Indication	A condition occurrence of Acute Coronary Syndrome<br>∧<br>Age ≥ 18 years<br>∧<br>Continuous observation of at least 0 days before and 0 days after index

Eligibility Criteria	No long-term LLT Use	Exactly 0 drug exposures to LLT[2] ∧<br>Exactly 0 measurement of Total Cholesterol > 240mg[3]<br>∨<br>≥ 1 drug exposures to LLT[2] ∧<br>Exactly 0 measurement of Total Cholesterol > 200mg[3]
	No Statin Use over 80mg	Exactly 0 drug exposures to Statins ≥80mg[1]
	No LLT Use with Fibric Acid Derivatives	Exactly 0 drug exposures to LLT with Fibric Acid[1]
	No LLT Use with Niacin	Exactly 0 drug exposures to LLT with Niacin[1]
	No CYP450 3A4 Use	Exactly 0 drug exposures Inhibitors of CYP450 3A4[1]
	No Percutaneous Coronary Intervention	Exactly 0 procedures of PIC[3]
	No Coronary Artery Bypass Surgery	Exactly 0 procedures of Coronary Artery Bypass Surgery[4]
	No Obstructive Hepatobiliary Disease	Exactly 0 diagnoses of Obstructive Hepatobiliary Disease[2]
	No Liver Disease	Exactly 0 diagnoses of Liver Disease[1]
	No Creatinine Kinase Levels > 3x Normal	Exactly 0 measurements of Creatinine Kinase > 354 U/L ∧ Gender is Female[1]<br>∨<br>Exactly 0 measurements of Creatinine Kinase > 318 U/L ∧ Gender is Male[1]
	No Creatinine Levels > 20mg	Exactly 0 measurements of Creatinine > 20mg[5]

[1]between all days before and 1 days before index start date
[2]between 365 days before and 1 days before index start date
[3]between 180 days before and 1 days before index start date
[4]between 60 days before and 1 day before index start state
[5]between 30 days before and 1 day before index start state

SUPPLEMENTARY TABLE 6: ACCOMPLISH, Concept Definitions, Indication

		Included Concepts		Excluded Concepts	
Indication	concept	concept code	concept name	concept code	concept name
	Hypertension	316866	Hypertensive disorder		

Note that all concept definitions include descendants

SUPPLEMENTARY TABLE 7: ACCOMPLISH, Concept Definitions, Eligibility Criteria

		Included Concepts		Excluded Concepts	
	concept	concept code	concept name	concept code	concept name
Eligibility Criteria	Antihypertensive Drug				
	ACE Inhibitors	21601783	Ace Inhibitors, Plain		
	Aldosterone	21601533	Aldosterone antagonists		
	Acute Coronary Syndrome	4215140	Acute coronary syndrome		
	Angina Pectoris	321318	Angina pectoris		
	Antihypertensive Drugs	21600381	Antihypertensives		
	Antidiabetic Drugs	21600744	Blood Glucose Lowering Drugs, Excluding Insulins		
		21600713	Insulins and Analogs		
		4336036	Oral Hypoglycemic Agents, Oral		
	Cardiovascular Disease (CVD)	134057	Disorder of cardiovascular system		
	Coronary Revascularization	37522318	Coronary artery bypass		
		4184298	Percutaneous transluminal angioplasty		
	Diabetes Mellitus	35502089	Glucose metabolism disorders (including diabetes mellitus)		
	Heart Failure	316139	Heart failure		
	Insulin	21600713	Insulins and Analogues		
	Ischemic Cerebrovascular Episodes	373503	Transient cerebral ischemia		
		36718067	Transient ischemic attack		
	Albumin	3024561	Albumin serum/plasma		
	Myocardial Infarctions	4329847	Myocardial infarction		
		35205189	Myocardial infarction		
	Overnight Fasting Plasma Glucose	3037110	Fasting glucose [Mass/volume] in Serum or Plasma		
	Peripheral Arterial Occlusive Disease (PROC)	2002187	Aorta-iliac-femoral bypass		
		37522314	Carotid endarterectomy		
		37522318	Coronary artery bypass		
		37520683	Leg amputation		
	Renal Disease	37019308	Renal disorder		
	Left Ventricular Hypertrophy	35205348	Ventricular hypertrophy	4231591	Right ventricular hypertrophy
	Serum Creatinine	3016723	Creatinine serum/plasma		
	Systolic blood pressure	3004249	BP systolic		
		3018586	Systolic blood pressure--sitting		
		3035856	Systolic blood pressure--standing		
		3009395	Systolic blood pressure--supine		
	Target Organ Damage	4349444	Hypertrophy, Left Ventricular		
		75650	Proteinuria		
		37019318	Renal failure		
		376103	Retinopathy		
		443605	Vascular dementia		
	Type 2 Diabetes Mellitus	201826	Type 2 diabetes mellitus		
	Unstable Angina	35205182	Angina unstable		

Note that all concept definitions include descendants

SUPPLEMENTARY TABLE 8: ACCOMPLISH, Cohort Creation

Indication	A condition occurrence of Hypertension<br>∧<br>Age ≥ 55 years<br>∧<br>Continuous observation of at least 0 days before and 0 days after index

Eligibility Criteria	High Systolic Blood Pressure or Treatment with Antihypertensives	≥1 drug exposures to antihypertensive drug[1]<br>∨<br>≥1 measurements of Systolic Blood Pressure ≥ 160mmHg[1]
	If Age ≥ 60 years, 1+ of the following; If 55 ≤ Age ≤ 60 years, 2+ of the following	
	Myocardial Infarction	a diagnosis of Myocardial Infarction[1]
	Unstable Angina	a diagnosis of Unstable Angina[1]
	Coronary Revascularization	A procedure of Coronary Revascularization[1]
	Stroke	A diagnosis of Stroke[1]
	Peripheral Arterial Occlusive Disease (PROC)	A diagnosis of PROC[1]
	Diabetes Mellitus	A diagnosis of Diabetes Mellitus[1]
	Left Ventricular Hypertrophy	A diagnosis of Left Ventricular Hypertrophy[1]
	Elevated Serum Creatinine	A measurement of Creatinine > 1.7[1] ∧ Gender is Male<br>∨<br>A measurement of Creatinine > 1.5[1] ∧ Gender is Female
	ACE Inhibitor or Aldosterone Receptor Blocker (ARB) Use with Elevated Albumin Creatinine Ratio	≥1 drug exposures to ACE Inhibitor/ARB drug[1] ∧ Albumin Creatinine Ratio > 300mg/dL[1]<br>∨<br>Exactly 0 drug exposures to ACE Inhibitor/ARB drug[1] ∧ Albumin Creatinine Ratio > 200mg/dL[1]
	No Angina Pectoris	Exactly 0 diagnoses of Angina Pectoris[2]
	No Heart Failure	Exactly 0 diagnoses of Myocardial Infarction[3]
	No Acute Coronary Syndrome	Exactly 0 diagnoses of ACS[3]
	No Coronary Revascularization	Exactly 0 procedures of coronary revascularization[2]
	No Stroke	Exactly 0 diagnoses of stroke[2]
	No Ischemic Cerebrovascular Episodes	Exactly 0 diagnoses of ischemic cerebrovascular episodes[2]

[1]between all days before and 1 days before index start date
[2]between 90 days before and 1 days before index start date
[3]between 30 days before and 1 days before index start date

SUPPLEMENTARY TABLE 9: RENAAL, Concept Definitions

		Included Concepts		Excluded Concepts	
Indication	concept	concept code	concept name	concept code	concept name
	Type 2 Diabetes Mellitus	201826	Type 2 diabetes mellitus		

		Included Concepts		Excluded Concepts	
Eligibility Criteria	concept	concept code	concept name	concept code	concept name
	Nephropathy	37019299	Nephropathy		
	Hypertension	316866	Hypertensive disorder		
	Systolic Blood Pressure	3018586	Systolic blood pressure--sitting		
	Insulin	21600713	Insulins and Analogues		
	Ketoacidosis	4209145	Ketoacidosis		
	HbA1c	40775446	Hemoglobin A1c | Bld-Ser-Plas		
	Pregnancy	44786908	HEDIS 2014 Value Set - Pregnancy Tests		
	Type I Diabetes Mellitus	201254	Type 1 diabetes mellitus		
	Non Diabetic Renal Disease	37019308	Renal disorder	443731	Renal disorder due to type 2 diabetes mellitus
				193782	End stage renal disease
				46271022	Chronic kidney disease
	Myocardial Infarction	4329847	Myocardial infarction		
		35205189	Myocardial infarction		
	Coronary Artery Bypass Grafting	37522318	Coronary artery bypass		
	Cerebrovascular Accident	36703451	Central nervous system haemorrhages and cerebrovascular accidents		
	Percutaneous transluminal coronary angioplasty (PTCA)	2000064	Percutaneous transluminal coronary angioplasty		
		4006788	Percutaneous transluminal coronary angioplasty		
	Transient Ischemic Attack (TIA)	373503	Transient cerebral ischemia		
	Heart Failure	316139	Heart failure		
	Renal Artery Stenosis	37003676	Renal vascular and ischaemic conditions		
	Primary Aldosteronism	35506454	Primary hyperaldosteronism		
	Phaeochromocytoma	4118993	Pheochromocytoma		

Note that all concept definitions include descendants

SUPPLEMENTARY TABLE 10: RENAAL, Cohort Creation

Indication	A condition occurrence of Type 2 Diabetes Mellitus<br>$\wedge$<br>70 years ≥ Age ≥ 31 years<br>$\wedge$<br>Continuous observation of at least 0 days before and 0 days after index

Eligibility Criteria	Nephropathy	A diagnoses of nephropathy exposures to antihypertensive[1]
	Hypertensive or Normotensive	A diagnoses of hypertension[1]<br>$\vee$<br>≥1 measurement of Systolic Blood Pressure ≥ 110 mmHg[1]
	No Recent Insulin Use	Exactly 0 drug exposures of Insulin[2]
	No history Ketoacidosis	Exactly 0 diagnoses of Ketoacidosis[1]
	HbA1c < 12%	≥1 measurement of HbA1c <12%[1]
	Not Pregnant	Exactly 0 measurements of a Pregnancy Test with value > 25[3]
	No Type I Diabetes Mellitus	Exactly 0 diagnoses of Type I Diabetes Mellitus[1]
	No Diabetic Renal Disease	Exactly 0 diagnoses of Non-Diabetic Renal Disease[1]
	No Myocardial Infarction	Exactly 0 diagnoses of Myocardial Infarction[4]
	No CABG	Exactly 0 procedures of CABG[4]
	No Cerebrovascular Accident	Exactly 0 diagnoses of Cerebrovascular Accident[2]
	No PTCA	Exactly 0 procedures of PTCA[4]
	No TIA	Exactly 0 diagnoses of TIA[5]
	No Heart Failure	Exactly 0 diagnoses of Heart Failure[1]
	No Renal Artery Stenosis	Exactly 0 diagnoses of Renal Artery Stenosis[1]
	No Primary Aldosteronism	Exactly 0 diagnoses of Primary Aldosteronism[1]
	No Phaeochromocytoma	Exactly 0 diagnoses of Phaeochromocytoma[1]

[1]between all days before and 1 days before index start date
[2]between 180 days before and 1 days before index start date
[3]between 300 days before and 0 days before index start date
[4]between 30 days before and 0 days before index start date
[5]between 365 days before and 0 days before index start date
